# Acute and long-term effects of chemotherapy on cognitive function among Indian breast cancer patients

**DOI:** 10.3332/ecancer.2025.1856

**Published:** 2025-02-25

**Authors:** Priti Singh, Chaithanya Leon, Simran Kaur, Atul Batra, Prashant Tayade, Muthukrishnan Suriya Prakash, Ratna Sharma

**Affiliations:** 1 Stress and Cognitive Electroimaging Laboratory, Department of Physiology, All India Institute of Medical Sciences, New Delhi 110029, India; 2 Department of Medical Oncology, All India Institute of Medical Sciences, New Delhi 110029, India

**Keywords:** chemotherapy, cognitive function, breast cancer, Chemobrain

## Abstract

**Introduction:**

Breast cancer (BC) is the leading cause of morbidity and mortality worldwide. Owing to early diagnosis and better therapeutic care, survivorship in these patients have improved tremendously. Chemotherapy, cornerstone in BC management have been associated with debilitating side effects including the effect on cognitive function, which significantly impairs the quality of life in these patients. Thus, it is imperative to understand the timeline and magnitude of the effects of chemotherapy on cognition to develop better management strategies. This is even more relevant in developing country like India, where there is inconspicuous absence of data in this regard.

**Aim:**

To study the acute and long-term effects of chemotherapy on the cognitive function in BC patients compared to chemotherapy naïve (Cx naïve) BC patients (disease controls) and matched healthy controls (HC) using subjective, objective questionnaires and neuropsychological tests (NPTs).

**Methods:**

The current cross-sectional study involved 120 participants, 30 each of Cx naïve BC patients, during chemotherapy BC patients (during Cx), post-chemotherapy BC patients (post Cx) and HC; all matched for age and education levels. Both subjective and objective assessments of cognitive functions were done in all the groups. Hindi Mental State Examination (HMSE) and FACT Cog questionnaire V3 were used for subjective assessment while Addenbrooke Cognitive Examination-III (ACE-III) questionnaire and domain specific computer based NPT (Wisconsin card sorting task (WCST) (learning), Flanker’s (attention and interference) and *n* back task (working memory) were done for objective assessment. The data were analysed for descriptive and inferential statistics, as appropriate using GraphPad Prism V9.

**Results:**

The subjective assessment using HMSE questionnaire revealed a significantly lower score in post Cx group as compared to HC (*p* < 0.001); however, it was comparable in other groups. FACT Cog V3 questionnaire revealed significantly higher cognitive impairment among those during Cx compared to Cx naïve patients (*p* < 0.001), post Cx BC patients (*p* < 0.001) and HC (*p* < 0.0001). Meanwhile, the objective assessment using ACE-III examination revealed significantly lesser scores among during Cx patients (*p* < 0.001), and post Cx BC patients (*p* < 0.0001) compared to HC group. In NPTs, WCST and *N* back working memory task revealed significantly lower accuracy in Cx naïve versus post Cx (*p* = 0.0054, *p* = 0.0068, respectively) and HC versus post Cx (*p* = 0.0054, *p* = 0.0045, respectively), while no significant difference was found in Flanker’s task. Furthermore, in WCST there were significantly higher scores present in total reaction time in post Cx compared to Cx naïve: *p* = 0.00444 and HC: *p *= 0.0003). In Flanker’s task reaction time was higher in all the groups (Cx naïve: *p* = 0.002, during Cx: *p* = 0.0007 and Post Cx: *p* < 0.0001) compared to HC. In addition negative correlation was found between the duration of chemotherapy with Perceived Cognitive Abilities (*r* = −0.482; *p* = 0.006), between the total number of cycles with Fact Cog Total (*r* = −0.373, *p* = 0.04) and Perceived Cognitive abilities (*r* = 0.39, *p* = 0.03), and between total dose and perceived cognitive abilities (*r* = −0.42, *p* = 0.014) also a positive correlation was seen between dose of epirubicin with reaction time of *n* back (*r* = 0.373, *p* = 0.04).

**Conclusion:**

Chemotherapy can have a negative impact on the cognitive function in BC patients, manifested as both acute and long-term effects, based on patient reported/subjective and laboratory based/objective cognitive function tests. The deficits were seen mostly seen in domains of attention and working memory across groups compared to matched HC.

## Introduction

Breast cancer (BC) is a leading cause of morbidity and mortality among women, constituting 11.7% of all reported cancers [1]. According to the global BC statistics report, it is now the most common malignant tumour worldwide, with an estimated 2.261 million new cases and 685,000 deaths worldwide in 2020 [1]. As per the Globocan data 2020, in India, BC accounted for 13.5% of all cancer cases and 10.6% of all deaths with a cumulative risk of 2.81% [2]. The prognosis of BC has significantly improved over time, with current 5-year survival rates of around 90% and 10-year survival at 80%, which makes it crucial to focus on enhancing survivorship in these patients.

Most of the BC patients receive treatment in the form of chemotherapy, wherein they are often plagued with numerous debilitating side effects. Chemobrain is also one of the consequences of chemotherapy-induced cognitive dysfunction/impairment seen in these patients. After treatment for BC, many patients complain of impaired memory, attention, speed of processing and other basic cognitive functions reflecting neurotoxicity due to chemotherapy [3]. The prevalence of chemotherapy-induced cognitive dysfunction is estimated to be 17%–75% of patients receiving chemotherapy [4–6]. Out of the affected patients, 17% to 30% appear to sustain long- 3 term cognitive impairment after Cx, even after Cx is over more than a year ago [7].

According to International Cancer Cognition and Cancer Task Force (ICCTF) most affected cognitive domains are learning, memory, processing speed and executive function by Wefel *et al* [8]. The committee developed recommendations for a core set of neuropsychological tests (NPTs), common criterion for defining cognitive impairment and cognitive changes and common approaches to improve the homogeneity of study methods in the patients of cancer receiving chemotherapy.

Among the regimens given, the routinely administered drug that is used for the treatment of BC are anthracyclines, particularly doxorubicin. Doxorubicin is a leading chemotherapeutic drug that can halt cellular replication and induce p53-dependent apoptosis in cancerous tissue. Although doxorubicin cannot cross the blood-brain barrier, but it potentially causes indirect neurotoxicity through neuroinflammatory mechanisms, possibly leading to cognitive deficits [9]. Furthermore, in a recent study by Vayyat *et al* [10] in Sarcoma patients, a positive correlation was found between of dose of doxorubicin and impairment in cognitive function.

The current study was designed to assess the acute and long-term effect of chemotherapy on cognitive function in BC patients on chemotherapy compared to chemotherapy naïve (Cx naïve) BC, Post Cx BC patients and healthy controls (HC) using subjective and objective questionnaires. To achieve this aim, we performed the subjective cognitive assessment using Hindi mental state examination (HMSE) [11] and Functional assessment of cancer therapy cognitive questionnaire version III (FACT Cog-V3) [12], and objective assessment using the Addenbrookes cognitive examination version III (ACE-III) [13] and computer-based NPT for various cognitive domains Wisconsin Card Sorting Test (WCST: learning, Flanker’s: attention, n back: working memory).

Furthermore, comparisons were made between the groups to see the effect of chemotherapy across both subjeive and objective cognitive assessments.

We hypothesised that there would be cognitive impairment in the patients during chemotherapy compared to Cx naïve, post chemotherapy BC patients and HC.

## Materials and methods

### Study design

**Figure 1.**
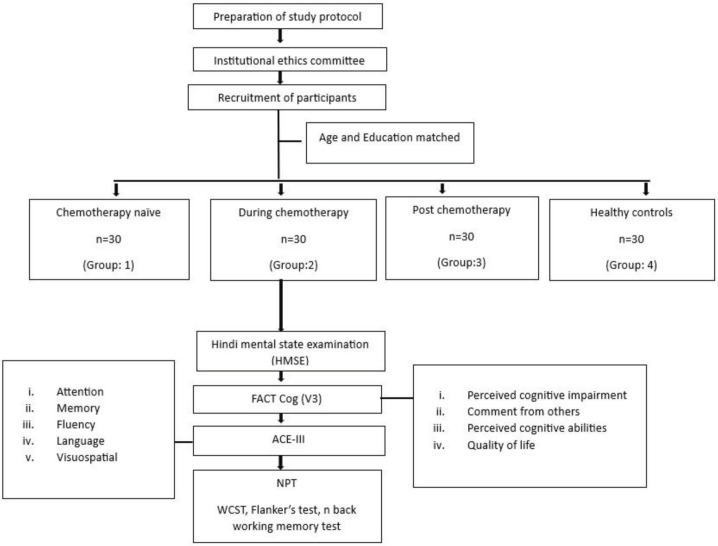
Flowchart describing the study protocol. After ethical approval, women diagnosed with BC and HC, in the age group of 18–65 years were recruited. Based on the chemotherapy status, the BC patients were divided in to three groups: Cx naïve, during chemotherapy- 3 months after the initiation of chemotherapy and post chemotherapy- 3 months after cessation of chemotherapy. Then the study participants were subjected to subjective assessments using HMSE and Functional Assessment of Cancer Therapy- Cognitive Function questionnaire Version 3 (FACT-Cog V3) and objective assessment using ACE-III. Further they performed NPT using WCST, Flanker’s test and n back working memory test.

This cross-sectional and observational study was conducted at the Stress and Cognitive Electroimaging Lab, Department of Physiology, All India Institute of Medical Sciences, New Delhi. After obtaining the ethical approval (Ref. No.-IECPG-203/24.03.2022), BC patients were recruited from the Department of Medical Oncology of Dr. B. R. Ambedkar Institute Rotary Cancer Hospital (Dr. B.R.A. IRCH), AIIMS, New Delhi, and matched HC were recruited on volunteering basis using advertisement, flyers and announcements in the community. The recruitment was done during the period from April to November 2022 (Figure 1).

### Participants

The study was performed on 120 subjects, 30 Cx naïve BC patients (recruited after the diagnosis of BC and before the cancer treatment (Group 1). 30 during Cx BC patients who had received at least 3 months of chemotherapy and come for follow-up within 7 days after the recent chemotherapy (Group 2) post Cx BC patients, who had completed their chemotherapy at least before 1 year (Group 3) (all BC participants, stage II–IV of Groups 2 and 3 received standard care of treatment comprising of anthracycline and Taxane for more than 3 months as mentioned in Table 1) and HC (Group 4). All the participants across groups were age and education matched and no confounding factors contributed to the matching of participants across groups. The participants recruited for Groups 1–3 had been clinically and histologically diagnosed with BC; they were age- and education-matched, had no history of neuropsychiatric or neurodegenerative disorders, and had no known brain metastases or central nervous system (CNS) lesions. Group 2 patients received a standard chemotherapy regimen consisting of anthracycline and taxane for more than 3 months, whereas Group 3 patients had finished the same regimen within the previous year. Group 4 consisted of age and education-matched females with no history of CNS lesions or neuropsychiatric/neurodegenerative disorders. The socio-demographic details are provided in Table 1.

### Procedure

After the recruitment of the participants for respective groups, written informed consent was obtained and detailed procedure was explained to them. Subsequently, socio-demographic and clinical history were collected and questionnaires were administered to each participant. For each participant, all the assessments were done on the same day and data were collected.

### Administration of questionnaires

#### Hindi mental state examination

HMSE was used as a global cognitive measurement questionnaire. There were 23 questions in HMSE and assessed orientation in time and place, memory, attention, concentration, recognition of objects, language function and comprehension and expressive speed. The total score of HMSE is 30, if the scores were above or equal to 24, it was considered as normal and below 24 was taken as cognitive impairment [11]. HMSE is better suited for neurocognitive testing of the largely Hindi-speaking Indian population and has a high sensitivity (0.81) and specificity (0.60).

#### Functional assessment of cancer therapy cognitive questionnaires (Fact Cog), version-3

It is a self-assessment scale validated in routine clinical practice to assess cognitive function in cancer patients [14]. It consisted of four subscales namely: perceived impairment (score range between 0 and 76), perceived abilities (score range between 0 and 28), comments by others (score range between 0 and 16) and impact on quality of life (score range between 0 and 16). The Cronbach's alpha for the scale for reliability analysis of the total scale and subscales were *α* ≥ 0.7 and ≥ 0.8, respectively [14].

#### Addenbrooke’s cognitive examination

It was used for the objective assessment of cognitive function for all the groups. It is composed of five cognitive domains: attention, memory, language, verbal fluency and visuospatial abilities [15]. It takes ~20 minutes to complete and has a maximum score of 100 (Attention = 18 points, Memory = 26 points, fluency = 14 points, language = 26 points, visuospatial skills = 16 points). Higher scores indicate better cognitive functioning. ACE-III also had good sensitivity and specificity for mild cognitive impairment with respect to Indian subpopulation, within a range of 0.83–1.00 at corresponding cut-off values of 84–89 [16]. Both Indian English and Hindi version of the 26 questionnaires are validated and free to use for clinical practice [17].

### Neuropsychological tests

The NPT were designed using E prime V3, as per the cognitive domains recommended by ICCTF [18]. The following cognitive function tests were employed:

#### WCST for learning and processing speed

**Figure 2.**
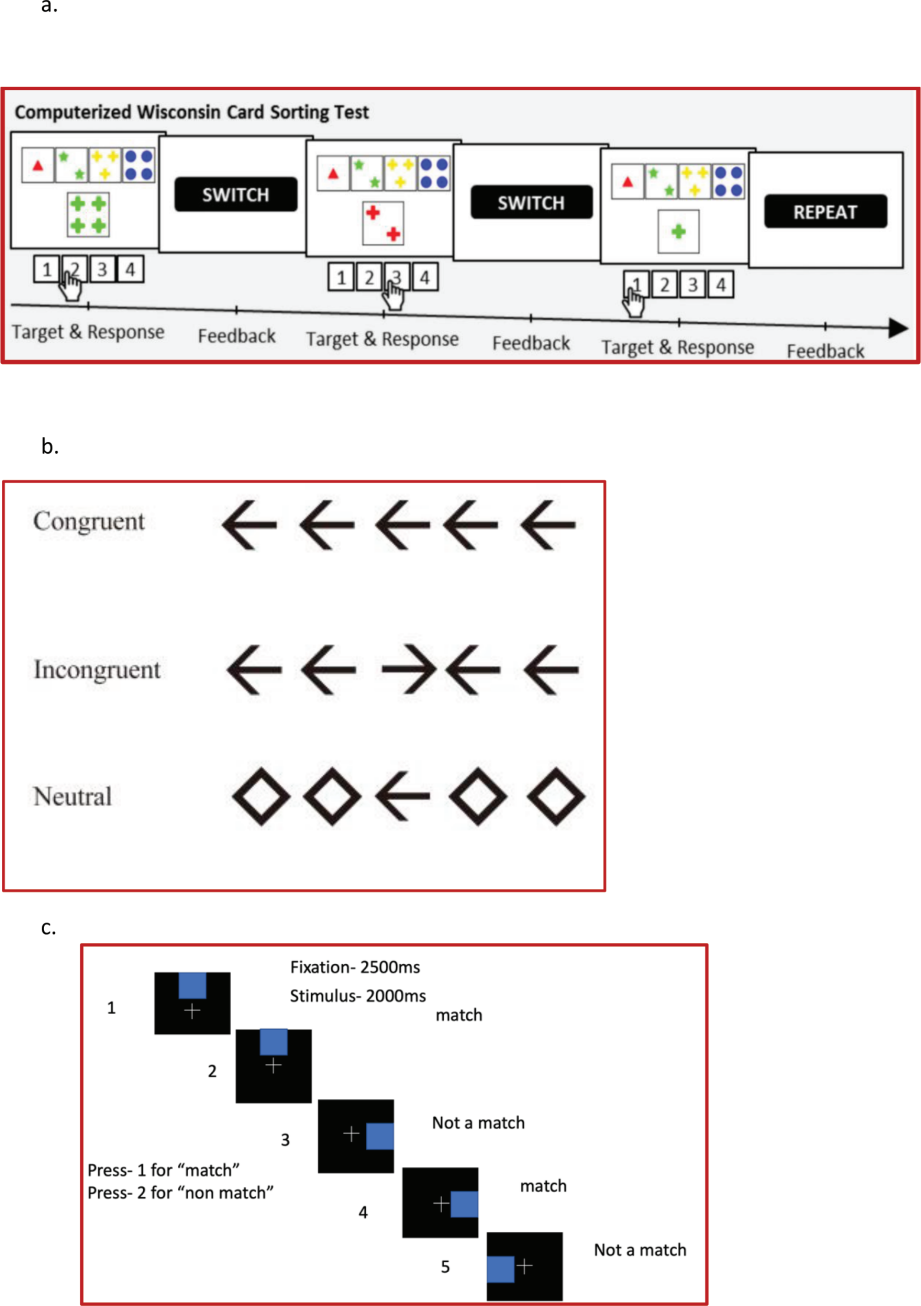
(a): Schematic representation of WCST, (b): Schematic representation of Flanker’s Task, (c): Schematic representation of n back working memory task.

The WCST comprised response cards with four types of geometric figures which differed based on form, number and color. In the WSCT, participants were required to apply sorting principles through trial and error [19]. The participants matched stimulus cards to four response cards every time without instructions from the test examiner. Participants were expected to deduce sorting principles according to feedback on correct or wrong matches and had to follow the sorting principle despite the changing conditions and neglect other unrelated geometric figures. To identify the prevailing category, participants had to rely on feedback that was provided during the task as ‘correct’ (positive feedback) or ‘incorrect’ (negative feedback) on each trial. The total number of trials were 60 and the behavioral parameters collected were reaction time, accuracy and a number of perseverative and non-perseverative errors and subsequently analysed (Figure 2a).

#### Flanker’s test for attention and processing

An arrow version of the Eriksen Flanker task was used for the current study [20]. During each trial, participants were presented with five black arrows for 200 ms against a white background. They were instructed to respond as quickly and as accurately as possible to indicate the direction of the middle arrow by pressing the respective buttons. A total of 60 trials were administered to the participants, comprising of congruent (Flanker’s pointing in the same direction as central arrow), incongruent (Flanker’s pointing in opposite direction to the central arrow) and neutral trials (Flanker’s were diamond that were not associated with response). A total of 60 trials were administered to all the four groups and responses were recorded through a button press. The behavioral parameters analysed were reaction time (total, correct and incorrect trials) and accuracy (Figure 2b)

#### n-back task for working memory task

Participants were presented a sequence of trial structures consisting of a blue colour small square, which can be in any of the eight possible locations of the big square. For every trial structure, the participants were required to respond to the current trial structure related to one presented one trial before (i.e., *n* = 1). The single trial comprised of the fixation cross for 2,500 ms followed by blue coloured trial structure, presented for 2,000 ms. In case the location of the subsequent blue square matches with the one shown just before, it is a match and the subject has to press 1; if not a match, the subject pressed the key 2. Behavioral parameters were ascertained in the form of reaction time and accuracy. A total of 60 trials were administered to all the subjects in the four groups (Figure 2c)

### Statistical analysis

GraphPad Prism 9.0 software was used to statistically analyse the demographic data and scores of the HMSE and FACT Cog-III and ACE-III questionnaires and NPT (WCST, Flanke’s test and n back working memory test). Kolmogorov- Smirnov test was applied to test the normality of the age distribution and education in years. Since the distribution was non parametric, Kruskal-Wallis test was used for comparisons between the groups. The correlation was done between the attributes of chemotherapy with questionnaire scores in Cx naïve, during chemotherapy group and post chemotherapy group using Spearman rho correlation analysis.

## Results

### Demographic and clinical characteristics

The study was performed on 120 subjects, 30 of each group. Table 1 shows the demographic profile of all the groups. Statistical analysis was done using GraphPad Prism 9 software. Kolmogorov-Smirnov test was applied to test the normality of the age distribution and education in years. It was found that there were no significant differences between the groups, i.e., the groups were age and education matched.

### Cognitive assessment using HMSE

The total scores of the post chemotherapy participants were significantly lower (*p* < 0.001) as compared to HC group while no significant difference was found in other group comparisons.

### Patient-reported cognitive assessment using FACT-Cog v3

The scores of FACT-Cog v3 questionnaire for each group are shown in Table 2. The total FACT- Cog score of the Cx naïve group was significantly higher than during Cx BC patients (*p* < 0.001) and significantly lower than HC (*p* < 0.05). Furthermore, scores in the chemotherapy group were lesser than post Cx (*p* < 0.5) and HC (*p* < 0.001).

### Objective neurocognitive assessment using ACE-III

The scores of (ACE)-III questionnaires for each group are shown in Table 3. The total ACE-III (*p* < 0.001) score along with memory and frequency domain scores (*p* < 0.01 and *p* < 0.05, respectively) were significantly lower in Cx naïve group as compared to the HC group. Furthermore, the total ACE-III scores of the during Cx group (*p* < 0.001) and post Cx (*p* < 0.0001) were significantly lower as compared to the HC group. We also observed the scores for language and frequency domain to be significantly lower in during Cx group compared to post Cx (*p* < 0.0001) and HC group (*p* < 0.0001).

### Objective neurocognitive assessment using NPT

The scores of WCST, Flankers task and *n*-back working memory task for each group are shown in Table 4.

**WCST:** The reaction time and non-preservative error of post Cx group was found to be higher than Cx naïve and HC (*p* = 0.0444 and *p* = 0.0003; *p* = 0.0288 and *p* < 0.0001, respectively). While, the accuracy score of post Cx was lower than Cx naïve, during Cx and HC groups (*p* = 0.0054, *p* = 0.0174 and *p* = 0.0054, respectively).

**Flanker’s task:** The reaction time was found to be higher in Cx naïve, during Cx, post Cx as compared to HC (*p* = 0.0002, *p* = 0.0007 and *p* < 0.0001, respectively), and while no significant difference was found in accuracy scores on between-group comparison.

***n*-back working memory task:** The reaction time of Cx naïve group was found to be higher than HC group (*p* = 0.0151) while, accuracy was found to be lower in Cx naïve as compared to HC and post Cx (*p* = 0.0123 and *p* = 0.0068, respectively).

### Correlation analysis

Correlation analysis was done between the total no. of the cycle of chemotherapy, total duration and total dosage of chemotherapy with cognitive function tests and questionnaire scores of during the chemotherapy group. Since the data were non parametric, Spearman rho correlation analysis was performed. A negative correlation was found between the duration of chemotherapy with perceived cognitive abilities (*r* = −0.482; *p* = 0.006) as well as between the total number of cycles with a total score of Fact Cog questionnaire (*r* = −0.373, *p* = 0.04) and perceived cognitive abilities (*r* = 0.39, *p* = 0.03). Also, between total dose and perceived cognitive abilities (*r* = −0.42, *p* = 0.014). While, a positive correlation was seen between the dose of epirubicin and with reaction time of *N* back task (*r* = 0.373, *p* = 0.04).

## Discussion

BC is one of the prevalent cancers with millions of women diagnosed each year across developed and developing countries. Due to the advent of newer treatments and better screening, the survivorship of these patients has improved. This calls for the need to focus on enhancing their quality of life by exploring the impact of chemotherapy [21]. One of the potential effects of chemotherapy is chemotherapy-induced cognitive deficits, also called Chemobrain [22]. The current study aimed to address the cognitive effects during chemotherapy in BC patients, across both subjective and objective domains. Concurring to the ICCTF [8], the core cognitive domains that were investigated in patients of BC (Cx naive), during Cx, post Cx as compared to matched HC were executive function, processing speed, attention and memory and learning.

Subjective assessment using HMSE in BC patients revealed comparable scores with HC. This is in line with Shen *et al* [23] but contrary to Liu *et al* [24] wherein BC patients who received chemotherapy had lower scores in MMSE compared to matched healthy subjects. Meanwhile, FACT Cog v3 questionnaire showed lower scores in during the chemotherapy (during Cx) group compared to post chemotherapy (post Cx), Cx naïve and HC. We also observed a decline in cognitive function domains of perceived cognitive impairment, comment from others, perceived cognitive abilities and quality of life in during the chemotherapy group compared to Cx naïve, post Cx and HC. Similar observations demonstrating the cancer-related perceived cognitive challenges were reported in BC patients, after three cycles of chemotherapy compared to baseline [25] and poor FACT cog scores within the chemotherapy group compared to Cx naïve patients [26]. Contrary to this, Lange *et al* [27] detailed no significant difference in subdomains of FACT Cog scale in Cx naïve versus post Cx except for perceived cognitive abilities.

Objective cognitive evaluation utilising ACE-III revealed lower scores found in during Cx and post Cx as compared to HC. The deficits were seen only in sub-domains of memory and visuospatial skills, language and frequency. This suggests that cognitive changes related to chemotherapy may not consistently affect all cognitive domains measured by the ACE-III. This underscores the significance of considering specific cognitive functions and their vulnerability to chemotherapy-related effects when assessing cognitive performance in cancer patients [11].

Objective assessment was also done using computer-based cognitive function tests. Post Cx displayed significantly higher reaction times, lower accuracy and increased non-perseverative errors compared to Cx naïve and HC utilising WCST. In line with our study, Cerulla *et al* [28] reported higher perseverative errors compared to pre-chemotherapy and 6-month chemotherapy groups. The performance using Flanker’s task showed no significant differences in accuracy, but Cx naïve, during Cx, and post-Cx displayed higher reaction time compared to HC. Contrary to what Swainston *et al* [29] reported, higher error rates in a chemotherapy group compared to a non-cancer group and no significant differences were reported in reaction time between the two groups. These findings highlight the complex nature of chemotherapy-related cognitive changes and emphasise the significance of assessing multiple cognitive domains to fully understand their impacts [29]. To assess, the effect on memory, *N*-back working memory task was used, wherein higher reaction time was seen in Cx-naïve individuals compared to HC, with lower accuracy in all BC groups compared to HC. McDonald *et al* [30] also reported no significant difference in reaction time and accuracy of groups between those who had completed 1 month of chemotherapy, completion 1 year after 1 month of chemotherapy and baseline (BC patient without chemotherapy utilising n back working memory task.

In addition, literature also reports chemotherapy patients display longer reaction times compared to HC, indicating potential delays in processing information among individuals undergoing chemotherapy. However, no significant differences were observed in accuracy between the chemotherapy group and the HC in our study. These discoveries further highlight the multifaceted nature of cognitive changes related to chemotherapy and emphasise the importance of examining various cognitive domains in understanding its impact [31]. A prospective study of patients with BC found significant cognitive decline soon after chemotherapy, which in some patients partially recuperated 1 year after treatment [32].

Furthermore, we found negative correlations between the duration of chemotherapy and perceived cognitive abilities, as well as between the total number of chemotherapy cycles and scores on the FACT Cog v3 total scale and perceived cognitive abilities. Additionally, a negative correlation was observed between the entire dosage of chemotherapy and perceived cognitive abilities. Also, a positive correlation emerged between the dosage of epirubicin and reaction time of *n* back task which is in line with a study by Vayyat *et al* [10] wherein correlation was found in cumulative dose of chemotherapy and cognitive functions.

The study's findings emphasise the need to create a care plan for these patients that allows for the monitoring of chemotherapy's side effects. This could support a potential modification to the BC treatment plan, specifically with respect to the total dosage and/or length of chemotherapy, thereby improving the quality of life in these patients.

## Strength and limitations

The present study describes the chemotherapy-related cognitive deficits in BC patients recruited from a tertiary health care centre. One of the strengths of the present study is the comprehensive evaluation of cognitive functions using both patients reported, subjective and objective assessment tools. Also, the participants in the study were recruited across 4 groups, matched in age, education and socio-economic status, especially in Indian settings, where there is sparse data in the domain of chemotherapy-related cognitive deficits.

Though the present study provides an important vision regarding the neurocognitive impairments caused by chemotherapy and cancer per se but it is limited by its sample size and further, a prospective longitudinal study would shed more light on the effects of chemotherapy on cognitive function, which authors plan to do in the future.

## Conclusion

The current study demonstrates the presence of cognitive impairment both on subjective and objective cognitive functions, in Cx naïve, during chemotherapy and post chemotherapy BC patients as compared to HC. These impairments persist even after the cessation of chemotherapy, thereby calling for a regular neuro-rehabilitative follow-up of these patients, to address the long-term effects of chemotherapy, support recovery and improve the overall quality of life in these patients.

## Conflicts of interest

The authors have no conflicts of interest to disclose.

## Funding

The authors received no funding for this study.

## Author contributions

**Priti Singh:** Material preparation, data collection, analysis, manuscript writing **Chaithanya Leon:** Data collection, analysis and manuscript writing.

**Simran Kaur:** Study conception, supervised data collection, analysis and manuscript revision.

**Atul Batra:** Contributed to the study conception and research design.

**Prashant Tayade:** Contributed to the study conception and research design.

**Suriya Prakash M:** Contributed to the study conception and research design.

**Ratna Sharma:** Contributed to the study conception and research design.

The first draft of the article was written by Priti Singh, and all authors reviewed and approved the final version.

## Data statement

The data which are in support of this study are available on request from the corresponding author. These data are not publicly available due to privacy and ethical restrictions.

## Figures and Tables

**Table 1.** Demographic characteristics of the study participants for all the groups.

	Group 1 Cx naïve (*n* = 30)	Group 2 During chemotherapy (Cx) (*n* = 30)	Group3 Post chemotherapy (Cx) (*n* = 30)	Group 4 HC (*n* = 30)
Age in years	42.97+/-8.90	47.20+/-8.19	47.47+/-9.08	41.83+/-9.07
Years of education	12.00 (9.50–16.00)	12.00 (7.00–17.00)	13.00 (9.00–15.00)	14.00 (12.00–15.00)
Educational status I. Post-Graduate II. Graduate III. Secondary School IV. Primary School	15% 18% 54% 13%	12% 16% 65% 7%	14% 18% 61% 7%	17% 16% 58% 9%
Stage of BC I. Stage ii II. Stage iii III. Stage iv	16.6% 56.66% 26.66%	13.33% 60% 26.66%	16.6% 53.33% 30%	Not applicable
Duration of chemotherapy in months	Not applicable	6.00 (3.750–7.00)	6.000 (4.00–7.250)	Not applicable
Number of cycles of chemotherapy	Not applicable	8.50 (4.00–10.00)	4.00 (4.00–4.00)	Not applicable
Dosage of epirubicin (units)	Not applicable	480 (447.5–523.0)	480.0 (360.0–520.0)	Not applicable
Total dosage of anthracycline (epirubicin) and taxane based (paclitaxel) (mg)	Not applicable	750.0 (430–1,213)	1,000 (720.0–1,960)	Not applicable

Demographic characteristics of the study participants for all the groups are represented as median (IQR). Educational status and stages of BC are represented as percentage (%) of participants in respective categories. Cx = chemotherapy

**Table 2.** The scores of the subjective cognitive symptoms from FACT-Cog v3 questionnaire.

FACT-Cog v3 subscales	Group 1 Cx naïve	Group 2 During chemotherapy (During Cx)	Group 3 Post chemotherapy (Post Cx)	Group 4 HC
Perceived cognitive impairments (CogPCI)	63.50 (58.50–66.25)**^!#^**	59.50 (55.75–61.25)^#*^	65.00 (60.25–68.00)**^#^**	67.00 (65.00–69.00)
Impact of perceived cognitive impairments on quality of life (CogQOL)	12.00 (10.00–14.00)**^!#^**	7.500 (5.750–8.250)^#*^	12.00 (12.00–15.00)	16.00 (15.00–16.00)
Comments from others (CogOth)	14.00 (13.00–15.25)**^#^**	12.00 (11.00–13.00)^#*^	15.00 (14.00–16.00)	16.00 (16.00–16.00)
Perceived cognitive abilities (CogPCA)	23.00 (22.00–26.00)**^#^**	22.00 (21.00–23.0)**^#^**	24.00 (22.00–26.0)**^#^**	26.00 (25.00–27.00)
Total FACT-Cog score	112.0 (106.8–117.5)**^!#^**	102.0 (94.75–104.3)^#*^	115.0 (107.8–120.3)	123.0 (116.3–126.0)

The table represents the scores of FACT-Cog v3 questionnaire for each group, expressed as median (IQR). *: denotes significant difference compared post chemotherapy group (*p* < 0.05); **#** denotes significant difference compared HC group (*p* < 0.05); !: denotes significant difference compared during chemotherapy group (*p* < 0.05). Cx = chemotherapy

**Table 3.** The scores of the objective cognitive assessment from the ACE-III.

ACE-III domains	Group 1 Cx naïve	Group 2 During chemotherapy (During Cx)	Group 3 Post chemotherapy (Post Cx)	Group 4 HC
Attention	15.50 (13.75–17.00)^*^	15.00 (13.00–16.25)^#*^	13.00 (12.00–14.00)**^#^**	17.00 (15.75–18.00)
Memory	23.00 (19.75–25.00)**^!#^**	19.50 (15.50–23.00)^#*^	23.00 (21.75–24.00)**^#^**	25.00 (23.75–26.00)
Fluency	8.00 (6.750–11.00)**^#^**	9.00 (7.00–10.00)**^#^**	9.00 (7.75–11.25)**^#^**	11.00 (9.00–13.00)
Language	24.00 (22.00–26.00)**^!^**	22.00 (21.00–24.00)^#*^	24.00 (22.00–25.00)	25.00 (23.75–26.00)
Visuospatial abilities	12.50 (11.00–14.00)	14.00 (12.00–16.00)	12.00 (11.00–14.00)**^#^**	15.00 (12.00–16.00)
Total ACE-III score	83.50 (75.50–90.00)**^#^**	80.00 (71.75–84.00)**^#^**	81.50 (77.50–86.25)**^#^**	90.00 (85.75–97.00)

The table represents the scores of (ACE)-III questionnaire for each group, expressed as median (IQR). *: denotes significant difference compared post chemotherapy group (*p* < 0.05); **#** denotes significant difference compared HC group (*p* < 0.05); !: denotes significant difference compared during chemotherapy group (*p* < 0.05). Cx = chemotherapy

**Table 4.** Behavioural scores (reaction time and accuracy) as assessed using objective neuro-psychological cognitive assessment tests.

Neuro-psychological tests	Parameter assesses	Group 1 Cx naïve	Group 2 During chemotherapy (During Cx)	Group 3 Post chemotherapy (Post Cx)	Group 4 HC
WCST	Reaction time (ms)	2,228 (2,008–2,708)^*^	2,196 (1,888–2,720)	2,801 (2073–3,666)	1,925 (1,809–2,215)^*^
	Accuracy (%)	90 (86.67–92.50)^*^	90.00 (84.17–92.92)^*^	85.00 (78.33–90.00)	90.00 (86.67–92.50)^*^
	Perseverative error	1.00 (0.0–3.250)	1.00 (0.75–3.00)	1.00 (1.00–5.50)	1.00 (0.00–3.00)
	Non-perseverative error	7.0 (4.00–9.250)^*^	6.500 (4.00–11.25)	8.00 (5.750–14.00)	5.00 (3.00–6.00)^*^
Flanker’s task	Reaction time (ms)	771.1 (578.2–939.5)**^#^**	717.6 (552.2–960.2)**^#^**	808.7 (649.9–1,007)	465.6 (436.7–528.4)^*^
	Accuracy (%)	95.87 (89.17–98.31)	95.00 (89.04–96.68)	95.00 (87.08–96.66)	95.87 (89.17–98.31)
N back working memory task	Reaction time (ms)	847.8 (620.0–1042)**^#^**	879.1 (698.4–1,021)	839.7 (553.6–1210)	637.7 (516.4–905.1)
	Accuracy (%)	58.06 (43.15–65.32)^#*^	53.23 (45.20–58.97)	49.17 (43.33–63.75)	59.68 (54.44–79.84)^*^

The table represents the scores of various neuro-psychological cognitive assessment tests for each group, expressed as median (IQR). *: denotes significant difference compared post chemotherapy group; **#** denotes significant difference compared HC group; denotes significant difference compared during chemotherapy group. Cx = chemotherapy
